# Multi-source data approach for personalized outcome prediction in lung cancer screening: update from the NELSON trial

**DOI:** 10.1007/s10654-023-00975-9

**Published:** 2023-03-21

**Authors:** Grigory Sidorenkov, Ralph Stadhouders, Colin Jacobs, Firdaus A.A. Mohamed Hoesein, Hester A. Gietema, Kristiaan Nackaerts, Zaigham Saghir, Marjolein A. Heuvelmans, Hylke C. Donker, Joachim G. Aerts, Roel Vermeulen, Andre Uitterlinden, Virissa Lenters, Jeroen van Rooij, Cornelia Schaefer-Prokop, Harry J.M. Groen, Pim A. de Jong, Robin Cornelissen, Mathias Prokop, Geertruida H. de Bock, Rozemarijn Vliegenthart

**Affiliations:** 1 University Medical Center Groningen, Department of Epidemiology, University of Groningen, Groningen, the Netherlands; 2 Department of Pulmonary Medicine, University Medical Center Rotterdam, Erasmus, Rotterdam, MC The Netherlands; 3 Department of Medical Imaging, Radboud University Medical Center, Nijmegen, The Netherlands; 4 Department of Radiology, University Medical Center Utrecht, Utrecht University, Utrecht, The Netherlands; 5 Maastricht University Medical Centre, Maastricht University, Maastricht, the Netherlands; 6 Department of Respiratory Oncology, KU Leuven-University Hospital Leuven, Leuven, Belgium; 7 Department of Clinical Medicine, University of Copenhagen, Copenhagen, Denmark; 8 Institute for Risk Assessment Sciences, Utrecht University, Utrecht, The Netherlands; 9 Department of Internal Medicine, University Medical Center Rotterdam, Erasmus, Rotterdam, MC The Netherlands; 10 Julius Center for Health Sciences and Primary Care, University Medical Center Utrecht, Utrecht University, Utrecht, the Netherlands; 11 Department of Pulmonary diseases, University Medical Center Groningen, University of Groningen, Groningen, the Netherlands; 12 Department of Radiology, University Medical Center Groningen, University of Groningen, Groningen, the Netherlands

**Keywords:** CT screening, Lung nodules, Lung cancer, Imaging biomarkers, Prediction model

## Abstract

Trials show that low-dose computed tomography (CT) lung cancer screening in long-term (ex-)smokers reduces lung cancer mortality. However, many individuals were exposed to unnecessary diagnostic procedures. This project aims to improve the efficiency of lung cancer screening by identifying high-risk participants, and improving risk discrimination for nodules. This study is an extension of the Dutch-Belgian Randomized Lung Cancer Screening Trial, with a focus on personalized outcome prediction (NELSON-POP). New data will be added on genetics, air pollution, malignancy risk for lung nodules, and CT biomarkers beyond lung nodules (emphysema, coronary calcification, bone density, vertebral height and body composition). The roles of polygenic risk scores and air pollution in screen-detected lung cancer diagnosis and survival will be established. The association between the AI-based nodule malignancy score and lung cancer will be evaluated at baseline and incident screening rounds. The association of chest CT imaging biomarkers with outcomes will be established. Based on these results, multisource prediction models for pre-screening and post-baseline-screening participant selection and nodule management will be developed. The new models will be externally validated. We hypothesize that we can identify 15–20% participants with low-risk of lung cancer or short life expectancy and thus prevent ~140,000 Dutch individuals from being screened unnecessarily. We hypothesize that our models will improve the specificity of nodule management by 10% without loss of sensitivity as compared to assessment of nodule size/growth alone, and reduce unnecessary work-up by 40–50%.

## Background

Lung cancer is one of the most frequently diagnosed cancers and the leading cause of cancer-related deaths worldwide in 2020 with an estimated 2.20 million diagnosed cases and 1.79 million deaths per year [1] The majority (about 82%) of lung cancer cases are attributable to smoking [2] and approximately 81% of lung cancer deaths in 2022 will be directly caused by cigarette smoking [3] Early detection through low-dose computed tomography (CT) has been proven to be a means of reducing lung cancer-specific deaths in a high-risk population. Lung cancer CT screening in long-term smokers reduced lung cancer mortality by 20–24% as supported by results from two trials: the Dutch-Belgian Randomized Lung Cancer Screening Trial (NELSON) and the US National Lung Screening Trial (NLST) [4, 5].

In NELSON, NLST, and other trials, all participants were regarded to be at high risk for lung cancer, but only a low percentage was diagnosed with lung cancer (0.7–2.2% at baseline). This has sparked discussions that if a cohort is invited for screening based on age and long-term smoking alone (like in NELSON), many individuals will not benefit. They may either have a low-risk for lung cancer despite their age and smoking history, or may have insufficient life expectancy to benefit from screening [6]. Thus, there is a critical need for better selection of individuals who will benefit from screening.

A drawback of NLST is the percentage of false positives. Over three screening rounds, 24.2% of CT scans were considered positive (lung nodule ≥ 4 mm diameter), but 96.4% of those were false-positives. The approach of the NELSON study, based on nodule volumetry and an intermediate test result category, led to a lower percentage of false positive results. Nevertheless, in NELSON, still one fifth of the participants had indeterminate or suspicious lung nodules at baseline [4], of whom 95.5% eventually tested negative for lung cancer. Thus, improved stratification and management of the detected nodules is needed, in order to prevent unnecessary follow-up screening and further diagnostic procedures.

In recent years, there has been an increasing recognition of the utility of risk models [7] to improve CT screening efficiency: to pre-select individuals with highest lung cancer risk in combination with sufficient life expectancy [6], and to evaluate the risk of lung nodule malignancy [8]. One study showed that PLCO2012, a model for participant selection based on education, body-mass index, family history of lung cancer, chronic obstructive pulmonary disease (COPD) and previous chest radiography in addition to age and smoking pack-years, showed higher sensitivity for lung cancer (83.0% vs. 71.1%) without loss of specificity, as compared to selection criteria based on age and pack-years alone (NLST data) [9]. Another study showed that the PanCan model outperformed the UKLS model in discriminating low- from high-risk lung nodules [area under the receiver operating characteristic (ROC AUC) 0.94 versus 0.58] in external validation [8].

There is great interest in evaluating new data sources to improve models for participant selection and nodule management [7]. Genetic and molecular markers, as well as CT-based biomarkers beyond lung nodules have been suggested [10, 11]. Very recently, air pollution as environmental factor was reported as another predictive factor for lung cancer [12]. Currently no validated risk prediction model incorporates such biomarkers or genetic susceptibility variants.

Technological advances have enabled the development of risk prediction models for lung cancer using multi-source data. Not only genetic and environmental data derived from the collected NELSON data, but also artificial intelligence (AI) based nodule evaluation, and chest CT imaging biomarkers beyond lung nodules may enrich prediction models and improve individual selection and lung nodule management.

The goal of this project is to improve the efficiency of lung cancer screening through expanding and integrating further NELSON data, addressing the limitation of previous approaches and developing multi-source risk prediction models for personalized risk assessment and lung nodule stratification. Therefore this project has the following objectives, namely to:

1. Construct and optimize polygenic risk scores for prediction of lung cancer.
2. Construct and evaluate air pollution-based environmental risk scores for prediction of lung cancer.
3. Determine the cancer probability of lung nodules using an AI risk score.
4. Measure CT biomarkers beyond lung nodules in NELSON screening rounds (emphysema, coronary calcification, bone density, vertebral height, body composition).
5. Develop and validate multisource data prediction models for selecting participants at the highest risk of lung cancer.
6. Develop and validate multisource data prediction models for lung nodule management with the aim to reduce the number of unnecessary follow up screenings and referrals.
7. Evaluate the cost-effectiveness of the newly developed prediction models.

## Materials and methods

**Fig. 1:**
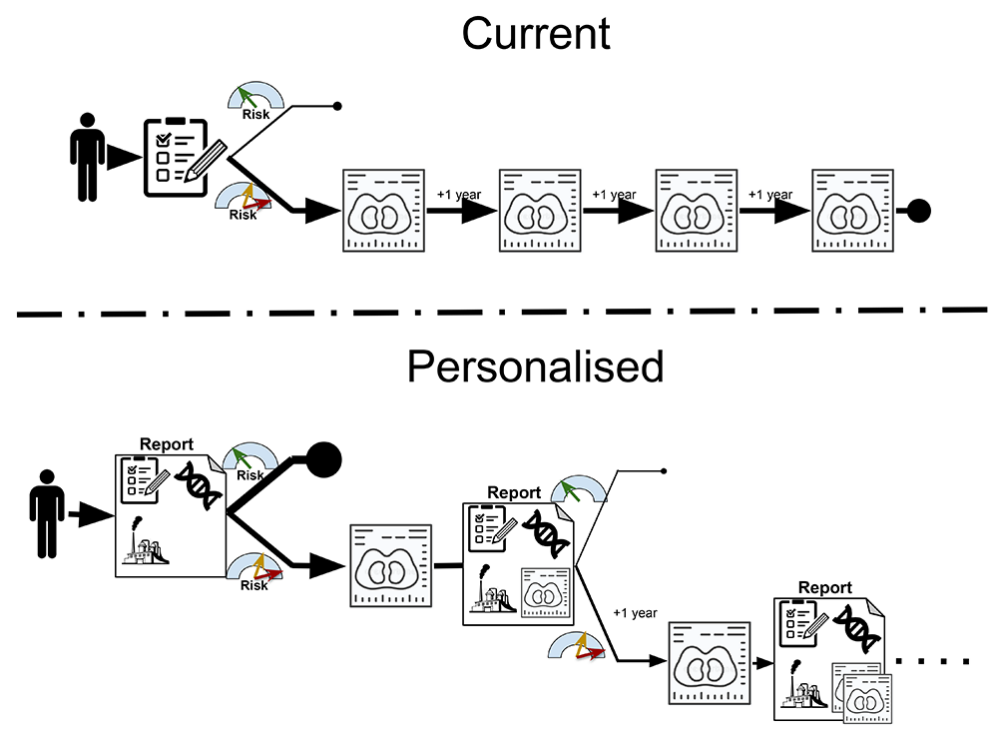
Screening strategy: top: current strategy, with same screen procedures for each screenee, and bottom: personalized approach implementing the multi-source data

This study is an extension of the NELSON trial with focus on personalized outcome prediction (NELSON-POP). New data will be derived for the NELSON study that have thus far not been considered in predicting lung cancer and survival. Based on already available and these new data, multi-source prediction models for pre-screening and post-baseline-screening participant selection and nodule discrimination will be developed to enable personalized screening strategies (Fig. 1).

### Population

The NELSON study is a Dutch-Belgian population-based, randomised controlled lung cancer screening trial. It has a follow up of 10 years after the screening rounds [4]. The study comprises 15,792 individuals 50–75 years old with a smoking history of over 15 cigarettes per day for more than 25 years or over 10 cigarettes per day for 30 years. Former smokers were included if they quit smoking ≤ 10 years ago. Participants were randomized 1:1 in a screening and control group. Subjects who were selected for screening were invited to undergo low-dose CT scanning of the chest at subsequent intervals of 1, 2, and 2.5 years. Participants filled out questionnaires at the start of the trial and at follow up after 10 years.

This project will also include data from the Danish Lung Cancer Screening Trial (DLCST) [13], a randomised trial with similar nodule management protocol as in the NELSON study. The DLCST compared annual CT screening for lung cancer with no screening in 4,104 individuals between the age of 50 and 70 years with a minimum smoking history of 20 pack-years, and had a follow-up of 5 years. The DLCST data will be pooled with the NELSON data for the evaluation of existing participant selection models and the development of the new model.

The results of this project will be externally validated using data of the 26,722 participants from the NLST CT arm aged 55 to 74 years, of whom 1701 were diagnosed with lung cancer [5].

### Data collection and analysis

**Fig. 2:**
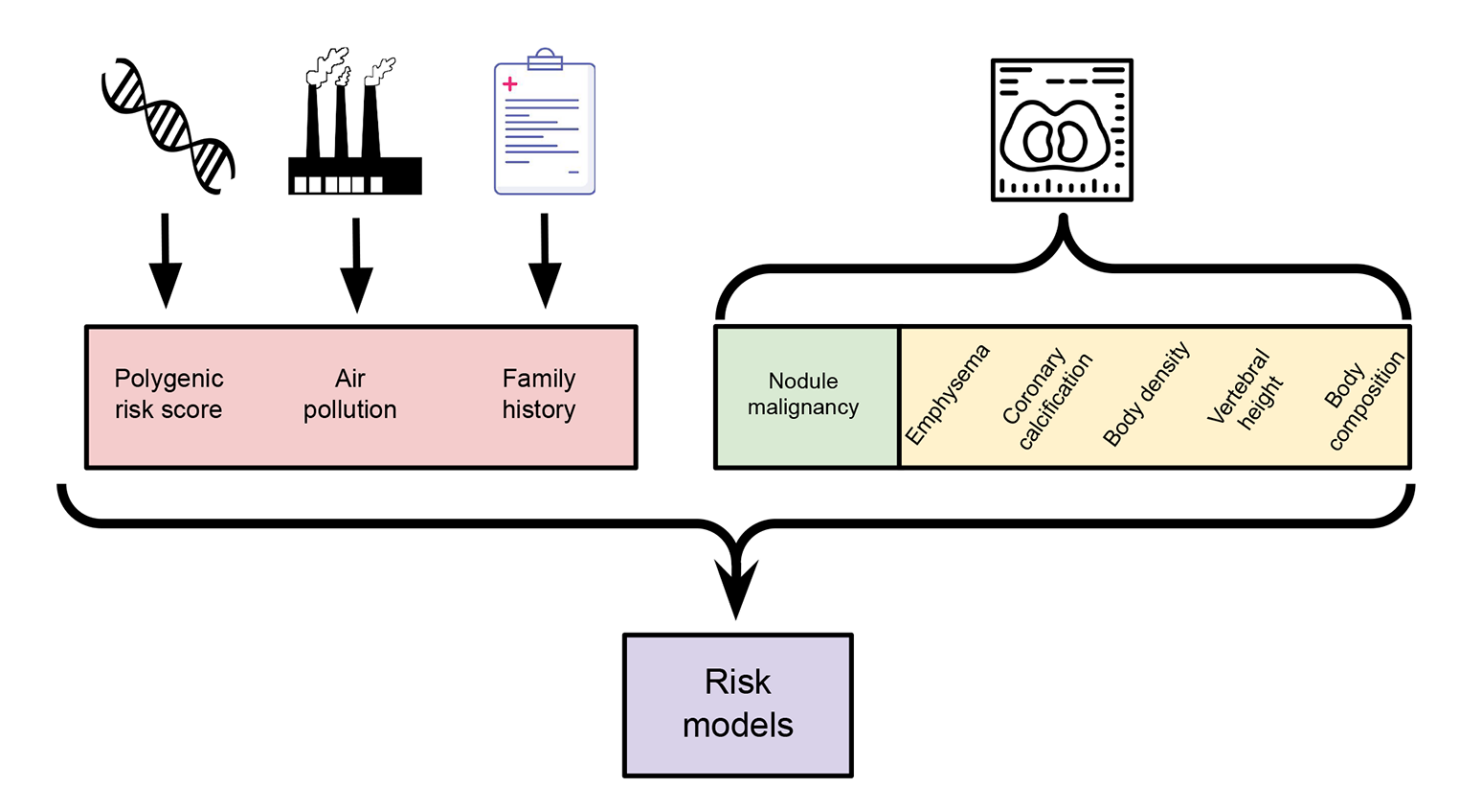
Multi-source prediction models that integrate genomic, environmental, imaging and individual characteristics data to select high-risk participants and high-risk lung nodules

Multi-source prediction models will integrate genomic, environmental, imaging and individual characteristics data to select high-risk participants and high-risk lung nodules (Fig. 2).

#### Genotyping

To address the 1st objective, genotyping of the NELSON CT-screening cohort will be completed. Of the NELSON CT screening cohort with blood sampling (N = 6,803), about 40% has previously been genotyped with arrays. DNA genotyping of the remaining 60% will be conducted. This effort will provide a comprehensive genetic profile of the NELSON CT screening cohort, which can be used to assess the role of various genetic variations, to construct polygenic risk scores (PRSs). For lung cancer, the genetic variants identified by the most recent genome-wide association study (GWAS) will be combined [463 Single Nucleotide Polymorphisms (SNPs)] to allow maximum predictive power [14]. Similarly, a PRS for lung emphysema, an important risk factor for lung cancer, will be constructed using available GWAS data (125 SNPs) [14].

After array genotyping, the dataset will be imputed to reference data (i.e. the 1000 Genomes resource [15] and/or the HRC haplotype reference consortium [16] and possibly TopMed [17] to harmonize, enhance and optimize the genotype content of the complete dataset. PRSs will be constructed for each participant by summing up all risk variants, weighted by variant effect size, as identified in prior GWAS studies. Raw PRS values will be z-transformed and used as a continuous predictor for lung cancer, using sex and smoking status as additional covariates. PRS performance will be expressed as an odds ratio per z-unit (standard deviation of the PRS) on lung cancer, as well as areas under the precision recall area under the curve (PR AUC) for continuous PRS, sex and smoking status, and a combined model. Additionally, risks of lung cancer in specific fractions of the PRS distribution relative to the average risk will be evaluated (e.g., cancer incidence in the highest/lowest 10% PRS of the NELSON population).

#### Environmental exposure

To address the 2nd objective, environmental data will be obtained, with a focus on air pollution that has demonstrated robust associations with risk of lung cancer. The following measures will be assessed: year average exposure (2000–2019) to particulate matter [with aerodynamic diameter ≤ 2.5 and ≤ 10 μm; particulate matter (PM)2.5 and PM10], ultrafine particles (≤ 100 nm), soot (i.e., PM2.5 absorbance), and nitrogen dioxide (NO_2_) [18].

Exposure to air pollution, as a measure of environmental risk, at the home address will be assessed. The collinearity of potential predictors will be evaluated and penalized regression as well as Bayesian multi-pollutant mixture modelling will be applied to estimate joint health effects. The predictive power of the environmental risk score for lung cancer and survival will be evaluated using Area Under Precision-Recall Curve (PR AUC).

#### Lung nodule malignancy

To address the 3rd objective, specific lung nodule features will be measured and an AI-based malignancy risk score will be computed for the lung nodules in the NELSON cohort, including baseline and new lung nodules. During the NELSON scan rounds, all CT scans were analyzed with the use of dedicated software (LungCare, version Somaris/5 VA70C-W, Siemens Medical Solutions). The analysis included semi-automated segmentation of all solid nodules, yielding quantitative imaging features such as nodule volume, diameter and average density. To complete the set of quantitative imaging features for nodules in NELSON, a novel semi-automatic algorithm for segmentation of subsolid nodules [19] will be applied to the set of subsolid nodules.

A previously published AI-based nodule malignancy estimation algorithm [20] will be used to compute the malignancy risk score for all baseline and new lung nodules in NELSON. To compare the AI-score against the performance of radiologists, an online observer study will be set up, in which radiologists will be asked to score the probability of lung cancer in a sample of NELSON lung nodules.

The performance of the AI-malignancy risk score will be compared to that of existing nodule risk models (Mayo Clinic model [21], PanCan model [22], UKLS model [23] and management guidelines for stratification, such as NLST [5], Lung-RADS, and the European Position paper [24]. The overall performance of the AI-risk score for lung nodule malignancy discrimination will be tested and compared with the performance of existing risk models for lung nodule discrimination, by measuring the AUC. Multi-reader, multi-case ROC analysis will be conducted to compare the readers score to the AI-risk score.

#### CT imaging biomarkers beyond lung nodules

To address the 4th objective, CT imaging biomarkers beyond lung nodules will be measured. Emphysema, coronary calcification, bone density, vertebral height and body composition will be assessed on baseline and incident round CT scans using AI-Rad companion Chest (Siemens Healthineers). Emphysema will be expressed by 15th percentile Hounsfield units (HU) of low attenuation voxels (Perc15) and the percentage of low-attenuation voxels below − 950HU. For coronary calcification, the calcium volume score will be measured. The CT density (in HU) of thoracic vertebrae will be used as measure of bone density; height of the vertebrae will be measured to assess osteoporotic fractures [25]. Body composition will be evaluated as areas of subcutaneous fat and muscle, that will be semi-automatically measured with in-house developed software [26].

In a subset of 250 subjects with a short-term repeat CT, AI-based segmentations of baseline and short-term repeat CT scans will be visually checked for accuracy. The AI-software will be run on the baseline and short-term interval scan to re-evaluate the biomarkers, and assess repeatability and inter-scan variability. Based on the results, rules will be derived for checking AI results in the remaining NELSON scans. In addition, randomly AI segmentations/results will be visually checked for every 1 in 100 scans.

#### Risk models

To address the 5th and 6th objectives, multi-source prediction models will be developed for (1) selecting participants at the highest risk of lung cancer for CT screening, and (2) optimized lung nodule discrimination. Both models will be developed for baseline and incident screening rounds using combined data from NELSON and DLCST studies where possible. For developing the model for participant selection at baseline, the data from self-report questionnaires, genetic and environmental risk scores will be used. For the incident rounds, the participant selection model will also include AI-risk score for lung nodule malignancy and CT imaging biomarkers beyond lung nodules. For developing the model for lung nodule discrimination at baseline, the aforementioned data for the participant selection model will be combined with AI-risk score for lung nodule malignancy and CT imaging biomarkers beyond lung nodules, as well as lung function results (available in a NELSON subset).

Machine-learning methods will be applied to (re)classify lung cancer risk and nodule discrimination after each round, using generated and existing participant/nodule information. Logistic regression will be compared with off-the-shelf machine-learning methods such as support vector machines and gradient boosted trees. To rank the classification models, we will use the average PR AUC from the outer cross-validation loop and compare the ${F}_{1}$ score as a secondary performance measure. Shapley values will be used to uncover the importance of the input variables to explain the predictions of the machine-learning models. The performance of the new model for participant selection will be compared with the NELSON inclusion criteria and with existing validated models for participant selection, including PLCOm2012, LLP, Bach model, LCRAT and Two Stage Clonal Expansion Incidence Model [9, 27–29]. The performance of the new lung nodule discrimination model will be compared to the original and updated NELSON model. The performance will be primarily evaluated by looking at changes in the PR AUC, and by precision, sensitivity, and specificity as secondary performance measures. To externally validate the new models, we will apply these on NLST data and evaluate the performance in predicting lung cancer and survival.

#### Cost-effectiveness model

To address the 7th objective, the cost-effectiveness of lung cancer screening based on the developed models will be evaluated. The SiMRiSc simulation model will be applied [30]. The outcomes will be: lung cancer mortality reduction, life-years gained, and incremental cost-effectiveness ratio. Tumor induction by the radiation from CT exposure will also be considered. The comparator will be the participant selection and screening efficiency as performed in the NELSON study.

In the cost-effectiveness model, one-way sensitivity analyses will be carried out to explore parameter uncertainty of the most cost-effective scenario at the assumed threshold per life year gained. The baseline values of the input parameters will be varied by an increase and decrease of two standard deviations for the base case analysis.

### Ethics statement

The original NELSON study was conducted with the approval of the Dutch Minister of Health after positive advice from the Dutch Health Council and by the Ethical Boards of the participating centers [4]. This study falls within the scope of the original informed consent in which side studies are allowed.

## Expected results

Of 7,135 participants in the NELSON CT-screening arm, 390 (5.5%) developed lung cancer over 10-years follow up. Combined with the DLCST data, the total number of screened participants is 11,239, of whom 490 (4.4%) were diagnosed with lung cancer during the screening rounds or during follow-up.

The following outcomes are expected to be obtained: polygenic risk scores and air pollution as measures of genetic and environmental risk, the AI malignancy score for all lung nodules, and other CT imaging biomarkers. All new measures will be related to lung cancer risk and survival, and predictive measures will be included in new participant selection and nodule discrimination models. The newly developed models will be applied on the combined data from the NELSON study and DLCST where possible. The new multi-source models for participant selection and lung nodule discrimination will be externally validated on the NLST data. The cost-effectiveness of these prediction models will also be assessed.

The first model this study aims to develop, is for stricter selection of screening participants based on risk of lung cancer and sufficient life expectancy. In the NELSON CT-screening arm, the vast majority (82%) of deceased participants died from other causes than lung cancer [4]. Analysis of NLST data showed 8 times lower lung cancer risk for participants at the lowest risk decile as compared to the highest risk decile [31]. One study showed that pre-screening selection based on life-years gained instead of risk, elevated life expectancy per screen-detected lung cancer by 7%, and reduced the number of screenees by 8.2% [32]. A risk model based on participant characteristics, compared to age and smoking pack-year criteria alone, prevented 20% more lung cancer deaths, combined with 17% decrease in number needed to screen [29]. The new model for participant selection using the data extracted within this project, such as air pollution and additional self-reported data, aims to reclassify the lung cancer risk and reconsider the decision to screen after each screening round.

This project will for the first time integrate CT features for post-baseline-screening participant selection to recalibrate the predictive model and determine whether continuing screening is of benefit. Based on estimates from previous studies [29, 31, 32], we hypothesize that the new model for participant selection will identify 15–20% participants with a low risk of lung cancer or short life expectancy, who will not benefit from lung cancer screening. This approach may prevent ~ 140,000 Dutch individuals from being screened unnecessarily, when lung cancer screening is implemented in the Netherlands.

To reduce the burden for those eventually selected for screening, a better method is needed to select CT-detected nodules with sufficiently high malignancy risk that would warrant short-term repeat CT or referral to a pulmonologist. In the NELSON study, 1.6% and 19.2% participants had positive or indeterminate results at baseline, respectively. Eventually, 95.5% of them tested negative for lung cancer. A study [8] showed that nodule characteristics combined with presence of emphysema (yes/no) in addition to nodule size improved the specificity of lung nodule discrimination by 8–10% (taken a recalculated NELSON specificity of 78–80%, this could increase the specificity to 88–90%). The new model combining the lung nodule information with the other data extracted within this project aims to better discriminate benign and malignant lung nodules at baseline and incident screening rounds. We hypothesize that our prediction model for lung nodule stratification will improve the specificity by ~ 10% without loss of sensitivity as compared to nodule size only, and reduce unnecessary work-up by 40–50%.

## Discussion

Although lung cancer screening has shown to be effective in saving lives, a better selection of participants and discrimination of detected pulmonary nodules is of utmost importance to save costs and reduce burden of the participants. The goal of this project is to develop multi-source risk prediction models for personalized risk assessment and lung nodule stratification, thus optimizing the efficiency of lung cancer screening. To achieve that goal, this project is the first focused on integrating multi-source data from different domains going beyond individual and lung nodule characteristics. It means that static (such as genetic) and dynamic risk markers (such as imaging, environmental and behavioral markers) will be integrated, not only for baseline screening (selection) but also for continued screening.

PRS is one of the risk markers having the potential for improving lung cancer risk assessment, that will be integrated in the multi-source model of this study. A recent study from the UK showed modest improvement for lung cancer discrimination when integrating a PRS into a risk model for lung cancer screening [33], but it was not aimed at lung cancer specifically. Another study developed a PRS for lung cancer consisting of 19 SNPs have shown promising stratification of low and high-risk individuals (two-fold increased risk), beyond known predictors [10]. Since recent large-scale GWAS has now uncovered > 100 genetic associations with lung cancer, improved lung cancer PRSs are under construction [14]. PRSs can also be constructed for several risk factors for lung cancer, e.g., lung emphysema or DNA repair defects, which could be added into an integrated PRS for lung cancer.

In this study, a detailed data on air pollution derived from the postcode data of participants will be used. Air pollution is another established marker of lung cancer risk [34], that will be integrated in this study. A pooled analysis of data from 7 European countries showed that PM2.5 exposure is related to lung cancer incidence. The report of Global Burden of Disease from 2017 showed that about 14% of all lung cancer deaths can be attributed to outdoor air pollution [35].

Imaging algorithms based on deep learning have great potential to perform more reproducible and more objective image pattern recognition as compared to radiologist evaluation of nodule characteristics visible with the naked eye, and thereby may increase the precision and consistency of lung nodule discrimination. This increased precision can be used to develop optimized follow-up protocols, leading to fewer unnecessary follow-up CTs and referrals in lung cancer screening, and possibly, to earlier referral of suspicious lung nodules. Several papers and high profile challenges have shown the potential of AI for lung nodule malignancy estimation [36–38]. Consortium partners of the NELSON-POP project have developed an AI algorithm for nodule malignancy estimation using a large dataset of lung nodules from the NLST [5]. The algorithm was externally validated using the baseline nodule dataset from the Danish Lung Cancer Screening Trial (DLCST) [13]. The AI algorithm outperformed the PanCan model and performed comparable to thoracic radiologists [20].

So far, CT-based lung cancer screening has mainly focused on lung nodule detection and management. However, imaging biomarkers beyond lung nodule assessment, related to emphysema, coronary calcification, bone density, vertebral height and body composition, may assist in discriminating risk of lung nodule malignancy and mortality, potentially enhancing its cost-effectiveness [39]. Moreover, these biomarkers may give insight about survival time to such extent that continued lung cancer screening may not be an effective option. Emphysema has been found to be an independent risk factor for development of lung cancer and for mortality [39]. In NELSON, the extent of emphysema on screening CT scans was found related to lung function decline and to the development of clinical signs of COPD [40]. The amount of coronary calcification as expressed in a calcium score is strongly related to cardiovascular events and mortality. Previous NELSON results showed that an increase in coronary calcium volume of 500 mm^3^ increased risk of cardiovascular events in 3 years by 46% [41], and that coronary calcium in lung cancer screening CT scans predicts all-cause mortality [42]. Smoking is a known, independent risk factor for osteoporosis. In a NELSON subset, CT-determined osteoporosis was shown to be an independent risk factor for all-cause mortality [43]: the adjusted hazard ratio for each 10 HU decline in bone density was 1.1 (1.0-1.2). Assessing subcutaneous fat and skeletal muscle might further improving participant selection in lung cancer screening CT scanning.

The application of automated AI algorithms for biomarker quantification reduces measurement variability and saves time, especially in large datasets such as the NELSON database [25, 44]. The Siemens AI-Rad Companion will be used for assessment of emphysema, coronary calcifications and bone measurements. Recent validation studies showed a strong correlation of AI-Rad Companion-based emphysema quantification with spirometry results in smokers with and without COPD [44], and between bone density measurements and osteoporosis assessment [25]; and coronary calcium measures showed good correlation to standards of reference [45]. For body composition, an in-house (UMCU) developed automated AI algorithm will be used. Although not (yet) CE-marked, we have performed these measurements successfully in non-contrast CT scans and now have measurements in more than 1000 subjects [26].

The results of this project will be crucial to contribute to a sustainable, accessible and affordable healthcare system if lung cancer screening is implemented. An efficient lung cancer screening program will potentially also reduce the use of expensive therapies, which thus may have a positive effect on the costs associated with lung cancer care overall [46, 47]. The front-runner position in lung cancer screening research and virtual research infrastructure, combined with the important data sources that will be added to existing lung cancer screening data within this project, will create an attractive environment for more researchers and companies to collaborate and use NELSON data. This will contribute to further research and optimization in lung cancer screening.
